# Role of Bioreactor Technology in Tissue Engineering for Clinical Use and Therapeutic Target Design

**DOI:** 10.3390/bioengineering5020032

**Published:** 2018-04-24

**Authors:** Clare Selden, Barry Fuller

**Affiliations:** 1 Institute for Liver and Digestive Health, Division of Medicine, Faculty of Medical Sciences, University College London, Royal Free Hospital Campus, Rowland Hill Street, Hampstead, London NW3 2PF, UK; 2 Department of Nanotechnology, Division of Surgery & Interventional Science, Faculty of Medical Sciences, University College London, London NW3 2QG, UK; b.fuller@ucl.ac.uk

**Keywords:** mechanotransduction, tissue engineering, cell signaling, in vitro model, bioreactor

## Abstract

Micro and small bioreactors are well described for use in bioprocess development in pre-production manufacture, using ultra-scale down and microfluidic methodology. However, the use of bioreactors to understand normal and pathophysiology by definition must be very different, and the constraints of the physiological environment influence such bioreactor design. This review considers the key elements necessary to enable bioreactors to address three main areas associated with biological systems. All entail recreation of the in vivo cell niche as faithfully as possible, so that they may be used to study molecular and cellular changes in normal physiology, with a view to creating tissue-engineered grafts for clinical use; understanding the pathophysiology of disease at the molecular level; defining possible therapeutic targets; and enabling appropriate pharmaceutical testing on a truly representative organoid, thus enabling better drug design, and simultaneously creating the potential to reduce the numbers of animals in research. The premise explored is that not only cellular signalling cues, but also mechano-transduction from mechanical cues, play an important role.

## 1. Introduction

For tissue engineering purposes, bioreactors are used in three ways: to enable, in vitro, a mimic of the state in which cells exist in vivo so as to understand normal cell and molecular physiology; to expand cells for potential clinical use, for example in gene and cell therapies, or to mimic a pathological state in order to study the pathophysiology; and to establish new therapeutic targets and test potential new treatments in a more realistic setting than simple in vitro conventional culture. Success in this area would also reduce the burden of use of animals in pharmacological testing.

There are several other uses of bioreactors, both on a micro- and larger scale; often, small- and micro-bioreactors are used in manufacturing to design new processes of production prior to full scale fabrication, and lab-on-a-chip applications. These, however, are not the subject of this review. Rather, this review will cover, in the most part, design of bioreactors that intend to address the functional mimics of an in vivo environment.

### Requirements for Bioreactor Design

Recreating the natural cellular niche using bioreactors is not trivial, and all impacts on cell behaviour must be considered. For example, there are complex stimuli in vivo that a cell may be exposed to, related to biochemical or metabolic cues on the one hand (chemical stimuli) and mechanical stimuli on the other. There is a likely interaction between these signals that will impact cell performance, so that for bioreactor design it is key to fully understand normal cell behaviour at the molecular level.

This is particularly relevant when the intention is to mimic a specific pathophysiology with the intention of promoting or testing new therapies.

In short, a bioreactor design should consider in vivo tissue structure, cellular organization, and cell survival, which will in turn influence the ensuing function, so the thought processes must start with the functional requirements; one size will never fit all. Some examples from biology include the performance of blood vessels depending on their role; for example, the make-up of a vein usually delivering low pressure flow at low shear that is responsible not only for flow but for heat dissipation, compared with an artery responsible for high flows, at much higher pressures, especially close to the heart, which are designed to have thicker musculature in vessel walls and to be more elastic to deal with greater pressures and pulsatile flow; these tissue structures are often anisotropic. To model these in a bioreactor, not only the correct cell type but also the mechanical structures capable of delivering the function is necessary. Another example would be a bioreactor to mimic solid tissues without, for example, liver and kidney, which, in contrast, are not dependent on the alignment of particular fibers for function; these are more mechanically isotropic.

The success of static culture reactors even with 3D constructs is often limited by mass transfer issues, with either a lack of nutrients to maintain the constructs or failure from a build-up of endogenous waste products. This arises because the only movement of solutes within the construct is concentration gradient-dependent and relies only on a diffusion mechanism, so that larger molecules move more slowly across a gradient than smaller molecules.

Today’s bioreactors usually contain 3-dimensional constructs of cells formed from a single phenotype; co-cultures of cells of different phenotypes, e.g., epithelial and endothelial; or epithelial and fibroblastic, or indeed a mixture of several cell types aimed at recreating the in vivo niche. Mass transfer is improved by making the bioreactors dynamic, using, simply, convection; this fluid flow facilities mass transfer. Some simple examples of these mixing bioreactors achieving the dynamic state are spinner flasks or rocking or wave form bioreactors. However, these are not really mimics of any system in the body.

## 2. Bioreactor Designs

### 2.1. Perfusion Bioreactors

Perfusion reactors, in contrast, simulate the in vivo environment more closely. The more successful microbioreactors are based on perfusion systems [1,2], some with simple downward or cross flow, and others delivering a microgravity environment. The latter achieves greater mass transfer; examples include rotating wall cell culture systems and fluidised bed bioreactors. Nonetheless, the flow must be optimised: optimal perfusion leads to improved, tissue-specific expression, whilst too much can impact not only on cell proliferation, but survival and function possibly by the removal of some important paracrine factors important for cell survival [3]. Crabbe et al. [4] utilised the rotatory cell culture system (RCCS), to improve reseeding of decellularised lung tissue with lung cells (C10) and bone marrow-derived mesenchymal stromal cells (MSCs) and to determine an effect on differentiation of the recellularised construct. They demonstrated improved proliferation and decreased apoptosis in this dynamic culture, as well as evidence of differentiation of the stromal cell component; authors speculate this improvement over 2D culture is mediated by the biomechanical force resulting from fluid shear, and the increased mass transfer of nutrients, oxygen, and waste-product dilution, and suggest an application in providing engineered lung tissue and understanding the transition of normal-to-fibrotic lung phenotype, prevalent in chronic obstructive pulmonary disease (COPD) and affecting more than 60 million people.

The choice of scaffold for the tissue construct will also impact on mass transfer. The thickness of some “artificial” substrates hinders mass transfer, and pore sizes may not reflect in vivo tissue organisation. Decellularised tissues may offer a better scaffold environment.

### 2.2. Oxygenation

Another element that is frequently forgotten in bioreactor design is the delivery of suitable oxygen tensions, especially in bioreactors utilising culture media as the nutrient supply, since oxygen diffusion into aqueous solutions is poor, in contrast to the oxygen-carrying capacity of blood normally perfusing the body. Whilst microbioreactors can overcome this to an extent by having thin layers of liquid in the fluid path, good control of oxygen provision and consumption is difficult. Improvements in fluorescent oxygen sensors have led to advances in this area, although when the perfusion fluid has high protein content, as seen, for example, in plasma, the technology is not sufficiently robust. Oxygen delivery in whole organ bioreactors has hampered successful use [5]; for example, the metabolic demands of cardiomyocytes and hepatocytes for oxygen differ (27.6 and 18 nmol oxygen·mg protein^−1^·min^−1^, respectively) and are not met by a diffusional supply of oxygen in thick tissue constructs. Alternative oxygen delivery systems may require the use of perfluorocarbons [6] or more physiologically red blood cells but not whole blood, as that may introduce immune components leading to a systemic inflammatory response.

### 2.3. Sheer Stress

Whilst the dynamic state is favourable, since it introduces a degree of shear stress by the very nature of the flow, this also has an impact on performance. In some tissues it is advantageous, for example, in blood vessels; in others, it may not represent the physiological state, e.g., in the liver blood flow through the portal vein is 1200 mL/min; however, the metabolic cells of the liver, the hepatocytes, are protected by the sinusoidal endothelial cell fenestrae that protect the hepatocytes themselves from shear. None but the most sophisticated of bioreactors can easily mimic that.

### 2.4. Mechanical Stimuli

The mechanical stimuli that impact cell physiology can be engineered into bioreactors in several ways. Essentially, these stimuli are achieved by enforcing a mechanical load on a tissue or cell construct. Such forces include compression, shear stress, stretch and compression, and pressure loads. It is clear from biology that each of these are reflected in body systems: muscles, blood vessels, ligaments, and tendons are all exposed to stretch loads in different ways. Bones encounter compression and torsion in normal physiology. A broken bone has two phases of healing: that which requires no movement and that which requires a load to encourage bone and muscle growth, i.e., tissues can adapt performance according to mechanical stimulation. The two laws governing such adaptation in hard and soft tissues, respectively, were described in the 19th century and still hold true today (Wolff 1892 and Davis 1867) [7,8].

### 2.5. Mechano-Transduction and Cellular Signalling

At a more cellular level, these loads lead to mechano-transduction of cellular signalling pathways. Examples include focal adhesions, cell to cell contacts, integrins and cadherins, and nuclear deformation. Such changes take from seconds to weeks depending on whether they are receiving a stimulus such as surface rigidity, “sensing” the local environment (milliseconds to seconds), altering gene expression (minutes to hours), or changing cell behaviour and function (days), or even influencing tissue development (weeks). Proteins involved in these processes include focal adhesion kinases and YAP (yes-associated protein), among many others.

An area of burgeoning research is the impact of viscosity and stiffness on cellular signalling; viscosity and stiffness also impose a mechanical load and affect cell morphology [9]. This too should be encompassed in microbioreactor design. As well as mechano-transduction impacting on signalling, downstream gene expression will be altered; the role of the directional loading force can influence protein binding on extracellular matrices and thus is also critical in bioreactor design. The shear stress forces should represent the mechanical environment of the original tissue [10].

Other mechanically induced stimuli can lead to tissue differentiation; an example is the fate of stem cells subjected to a load, known as mechano-differentiation [11,12,13,14].

Stretch has most often been described in the context of vascular tissue engineering, and is effectively described as the “new” length divided by the initial length. Directional change can be in any direction, and the cyclic stretch observed in muscles leads to enhancement of protein expression and ecm protein content [15]. Often, the result of such stretch forces in bioreactor design is cell alignment that better represents the normal in vivo tissue environment. Cardiac tissues constructed under mechanical stretch and/or electrical stimuli display propagation speeds similar to those observed in vivo and respond to electrical stimuli by synchronised contractions [16,17].

### 2.6. Examples Used in Tissue Engineering and Pathophysiological Studies

Burk et al. [18], using decellularised tendons reseeded with mesenchymal stromal cells, applied mechanical stimulation with a cyclic-strain bioreactor. Natural horse tendon movement is best represented at a frequency of 1 Hz, which these authors used, and a strain of 2% was applied as a close estimate of that seen in the superficial digital flexor tendon in horses, and is comparable to that of the Achilles tendon in man. Stepwise time increases in strain and rest periods were implemented. Their data indicated cell anisotropy when comparing cells grown on scaffolds rather than monolayer, and increased differentiation; however, a negative impact on cell viability possibly arising from poor cell adaptation to the strain/rest cycles re-iterates the importance of the design elements of the bioreactor.

Examples of pressure in vivo are well described: atmospheric pressure on skin, i.e., a load distributed over an area, usually uniformly. Groeber et al. [19] devised a bioreactor to produce a vascularised skin construct that was comprised of 2 cell types initially in a fluid circuit in a BioVacSc. The perfusion flow produced 10 mmHg, initially rising to 80 mmHg, prior to the typical physiologic pulsatile pressure profile with systolic at 120 and diastolic 80 mmHg, respectively. Two fluidic circuits were added to deliver fluids to both the surface and underside of the construct for 6 days. Thereafter, the addition of human embryonic kidney (hEK) cells to the surface of the construct completed the model. Changes to the nutrient media and a switch to air-liquid interface conditions for 27 days revealed a well-stratified epidermis with appropriate structural layers and a strong epidermal barrier. The most important parameters were recreated in the bioreactor, and this model should enable studies on the interaction of cellular and non-cellular blood compartments with the dermal layers, which are useful for immunological research and with clinical potential for deep wounds.

### 2.7. Stretch/Compression

Arteries are subjected to blood pressure loads, and heart valves are subjected to alternating pressures, so that the valves receiving a pressure gradient signal respond by opening and closing. Clearly, this is a complex pattern of events, and when reproduced in bioreactors it enables tissue constructs that are closely aligned with tissues in vivo.

Egger et al. [20] demonstrated a parallel perfusion bioreactor, in which 8 conditions could be compared simultaneously under physiological shear stress and hydrostatic pressures necessary for differentiating osteogenic tissues from mesenchymal stem cells. The authors noted that when using an artificial scaffold the mechanical cues of fluid sheer stress led to sheer forces (10–40 mPa), an order of magnitude lower than observed in vivo, typically 0.3–3 Pa [21]. The porosity and pore sizes of the scaffold were responsible for the fluid sheer stresses that impacted cell and protein deposition; under dynamic flow expression of alkaline phosphatase, an estimate of osteogenic differentiation was highest, although hydrostatic pressure was not influential.

Compression is related to stretch in as much as it is directional but the opposite in force outcome: stretch leads to a greater than initial, and compression a lesser than original, dimension, as evidenced in nature by, for example, weight-bearing joints and cartilage such as the knee joint. Bioreactors mimicking compression models result in more ordered structures and increased mucopolysaccharide content of the extracellular matrix [22,23,24]. Even blood vessel lumens experience compression, but in a radial direction resulting from changes in blood pressure. The cellular reactions follow a force at two levels; the macroscopic force event leads to changes at the microscopic level due to the shear induced by the fluid flows from the tissue under compression.

In physiology, all of these forces may be acting together in a particular tissue, in, for example, arteries, heart muscle, and musculoskeletal tissues. Only bioreactors designed to mimic all of these forces including bending and torsion will provide a good model for studying normal and pathophysiological production of tissue constructs for clinical application and testing of potential therapeutic modalities.

### 2.8. Cell Seeding

The manner in which cells are seeded into bioreactors will also impact considerably their performance; in many cases, the intention is for cells to be uniformly seeded throughout the scaffold. Thus, pore size and the model of entry for cells will play an important role, not only during seeding but also for nutrient/metabolite and gas exchange during subsequent culture. Whilst simply applying a cell suspension to scaffold enables some cell attachment, and even infiltration deeper into the scaffold pores, it is an uncontrolled process and thus subject to considerable variability. Approaches that use different physical processes to drive the processes are better, such as acoustic or electromagnetics energy or even vacuum application [25,26,27].

### 2.9. In Silico Modelling

Since there is considerable complexity required in bioreactor design to mimic in vivo organ function, utilising computational modelling (in silico) from knowledge of normal physiology prior to bioreactor construction may improve outcomes. Tresoldi et al. [28] utilised a computational model with fluid-structure interaction to demonstrate the necessary parameters for vascular tissue engineering. Using the model in the MiniBreath bioreactor (pulsatile perfusion), it predicted pressures acting on the tubular scaffolds, circumferential deformation, solid components, and wall shear stresses, with good comparability with the analytical model. The model could not take account of scaffold thickness along the length, nor predict any changes associated with cell growth and maturation, thus emphasizing the importance of defining the key questions prior to modelling and appreciating the limitations of modelling versus analytical study. Nonetheless, for a complex system, much information can be gleaned form an in silico approach.

### 2.10. Scaffolds Used with Bioreactors

The choice of scaffold for tissue constructs, be it synthetic polymer materials (e.g., poly lactic acid, poly caprolactone, or polyglycolic acid), bioceramic-based or natural polymers (e.g., collagens, dextrans, gelatins, alginates, hyaluronic acids etc.), has the aim of aiding cell survival, function, and proliferation whilst concomitantly minimising immune responses, which is especially important for tissues to be used in clinical applications, such as heart valves. These scaffolds have been widely reviewed elsewhere [29,30,31,32,33,34,35,36,37,38,39,40,41], including their use with bioreactor technology. Typically, they are porous to increase cell/scaffold area and promote 3-dimensional growth of the seeded cells. A more recently exploited scaffold principle is that of using decellularised natural organs generated to maintain extracellular matrix components but be devoid of cellular DNA or protein (see following sections). Since there are no cellular components left on decellularised scaffolds, there should not be species cross reactivity, so that, for example, decellularised porcine organs could potentially be used for recellularisation with human cell material; nonetheless, the extracellular matrices of different species do indeed differ, so they may introduce incompatibilities, depending on the performance requirements. The choice of scaffold is likely to be key to producing a bioreactor system that achieves its aim, and several bioreactors incorporate such scaffolds.

The next sections will consider some case studies of bioreactors incorporating some or all of the above principles.

## 3. Bioreactors Used to Provide Tissue Constructs for Implantation

Ma et al. [42], utilising decellularised aortae derived from foetal pigs and seeded with canine endothelial cells for three days in a principally static environment to encourage cell attachment, were subjected to dynamic bioreactor culture for a further 7 days prior to implanting into carotid arteries. The bioreactor imposed a liquid flow starting at 20 mL/min, which gradually increased daily by 10 mL/min up to 60 mL/min; both static pressures (10 mmHg) and dynamic pressures (60 mmHg) were imposed as would occur in vivo. Six months after implantation, the grafts had remodelled, appearing as normal arteries with complete endothelial cell layers arising from those implanted, those native to the animal, and those from endothelial progenitor cells originating from the blood.

Whilst Ghaedi et al. [43] generated bioengineered lung tissue from induced pluripotent stem cell (iPSC)-derived epithelial cells on decellularised lung tissues from rat and human lungs, they emphasized the importance of good gas exchange across the lung, requiring integrity of the physical barrier expressed in vivo from both epithelial and mature endothelial cells with appropriate tight junctions and adhesive molecules. This study used only the epithelial cell component. Nonetheless, this example indicates that both within and across species decellularised scaffolds support the survival proliferation and function of airway epithelial cells.

The need to provide a shorter and more effective route for patients requiring a coronary artery bypass has led to tissue engineering efforts to produce small diameter vascular grafts of less than 6 mm diameter. This is a lengthy process; Tondreau et al. [44] designed a simple approach that reduced the time from more than 4 months to 4 weeks by starting with conventionally cultured fibroblast sheets that could be produced “offline”, that were subsequently rolled onto mandrels, decellularised, and recellularised with patients’ own endothelial cells in a perfusion bioreactor. Significant mechanical testing of the graft for burst pressure compliance, thickness, and suture strength retention (26 +/− 2 gf, compared with 138 +/− 50 gf) established a graft composed only of human dermal fibroblasts, reseeded with endothelial cells, which could shorten the timeline for patient treatment significantly.

An endothelial layer on biological graft matrices is considered important from the perspective of antithrombotic activity [45] and preventing graft failure. However, in artificial grafts, the pulsatile flow of a bioreactor can disrupt the endothelial cell surface under high flow conditions, as may be experienced by cusps during valve opening in the native valves, so that whilst one may endeavour to mimic the natural environment, some compromise in bioreactor designs may be required to enable adaptation of the recellularised grafts [46]. In a different organ system, the liver, again using decellularised tissue as the bioreactor scaffold, Hussein et al. defined a heparin-gelatin mixture as an antithrombotic agent prior to cell seeding that positively impacted attachment and migration of endothelial cells, as well as leading to enhanced function from the parenchymal fraction of subsequently seeded HepG2 epithelial cells [47].

Due to a shortage of organs for transplant, the research endeavour to “grow” organs for transplantation, and improvements in the decellularisation/recellularisation strategies, there is potential in this area. The bioreactors required to implement this approach must be specific for that organ’s physiology (for example, for the type of flow required, whether continuous perfusion or pulsatile flow [48]).

## 4. Bioreactors Designed for Disease Modelling

Tumour cell modelling for testing of new drugs in human cells, equivalent to tumour biopsies, would be a significant advance. Nietzer et al. [49] produced such a bioreactor using a jejunal decellularised scaffold and colon cancer cells with fibroblasts designed to have tissues at the interface of two separate fluid circulations meeting apical and basolateral-specific culture conditions: contemporaneously modelling optimized fluid circulations in terms of ambient pressures, inlet and outlet velocities, and defining shear stress conditions. The resultant tumour-like tissue expressed beta catenin at cell borders and had a stroma positive for vimentin and cytokeratins, which is typical of colon adenocarcinomas. This model was clearly delineated from the same cells as monolayer cultures by the 5FU (5-fluorouracil) response and exemplified the treatment response in man. There are several examples in the literature.

## 5. Small Bioreactors to Mimic Larger Production Bioreactors as Bioartificial Organs

Bioartificial organs not intended for transplantation but for temporary replacement of function have been developed [50,51,52,53], in particular for the liver system, since the liver, being highly regenerative, can repair itself given time after an insult of acute liver failure. Whilst organ transplants are curative, the lack of donor organs results in many dying before they receive a transplant. Several experimental models exist, and a few are in clinical trials. However, from a bioreactor perspective, culturing on a human scale does not enable rapid prototyping or optimisation of production conditions. Our own group has produced a bioartificial liver machine (BAL) on human scale based on a fluidised bed bioreactor design, which maximises mass transfer (UCLBAL), and tested it in a porcine model of acute liver failure [50,52,53]. To refine certain aspects, there was a need to develop a small scale mimic on the small (~30–50 mL) (not micro) scale but to nonetheless enable metabolic, gene expression, and protein studies, and since this small fluidized bed (sFBB) bioreactor enables flow studies that can be mathematically modelled, it is more easily scalable to clinical size and enables a comparison of dynamic versus static conditions [54] suitable for drug biotransformation. Multiple units can be run simultaneously, which increases research capabilities.

## 6. Conclusions

Over the past decade, significant improvements in design and construction of bioreactors have been made. Systems have been developed that allow robust and reproducible culture conditions to be maintained. Specific bioreactor design is critical to the production of useful systems that can predict performance if based on a natural cell niche from in vivo physiology. Whilst the more sophisticated the bioreactor approach, the more likely it is to reflect the natural physiological state, simpler designs are likely to be more operationally robust, so a compromise based on bioreactor complexity versus the essential functional parameters of the desired end-product will always be necessary.
